# The SuperChip for microbial community structure, and function from all environments

**DOI:** 10.1111/1751-7915.12045

**Published:** 2013-03-06

**Authors:** Terry C Hazen

**Affiliations:** 1 Departments of Civil & Environmental Engineering, Earth & Planetary Sciences, Microbiology, University of TennesseeKnoxville, TN, 37996, USA; 2 Biological Sciences Division, Oak Ridge National LaboratoryOak Ridge, TN, 37831, USA

## Abstract

**Summary:**

We have the technology and capability to develop an all-in-one microarray that can provide complete information on a microbial community, including algae, protozoa, bacteria, archaea, fungi, viruses, antimicrobial resistance, biotoxins and functional activity. With lab-on-a-chip, nanotechnology integrating a variety of the latest methods for a large number of sample types (water, sediment, waste water, food, blood, etc.) it is possible to make a desktop instrument that would have universal applications. There are two major thrusts to this grand challenge that will allow us to take advantage of the latest biotechnological breakthroughs in real time. The first is a bioengineering thrust that will take advantage of the large multidisciplinary laboratories in developing key technologies. Miniaturization will reduce reagent costs and increase sensitivity and reaction kinetics for rapid turnaround time. New and evolving technologies will allow us to port the designs for state-of-the-art microarrays today to completely new nanotechnology inspired platforms as they mature. The second thrust is in bioinformatics to use our existing expertise to take advantage of the rapidly evolving landscape of bioinformatics data. This increasing capacity of the data set will allow us to resolve microbial species to greatly improved levels and identify functional genes beyond the hypothetical protein level. A cheap and portable assay would impact countless areas, including clean water technologies, emerging diseases, bioenergy, infectious disease diagnosis, climate change, food safety, environmental clean-up and bioterrorism. In my opinion it is possible but it will require a very large group of multidiscplenary scientists from multiple institutions crossing many international boundaries and funding over a 5-year period of more than $100 million. Given the impact that this SuperChip could have it is well worth the price!!!

We have the technology and capability to develop an all-in-one microarray that can provide complete information on a microbial community, including algae, protozoa, bacteria, archaea, fungi, viruses, antimicrobial resistance, biotoxins and functional activity. With lab-on-a-chip, nanotechnology integrating a variety of the latest methods for a large number of sample types (water, sediment, waste water, food, blood, etc.) it is possible to make a desktop instrument that would have universal applications. With the advent of next-generation sequencing, for the first time we have sufficient DNA sequence information to begin to understand the complex interactions in environmental microbial communities. While sequencing billions of base-pairs from isolated genomic DNA of selected communities has given us a new understanding of the diversity and shear numbers of organisms involved in key environmental processes, the cost of a single well-characterized community is still well out of the reach of any but the largest sequencing centres. Additionally, sequencing is serial. That is, an individual DNA molecule is analysed in a sequencing reaction. New technology has allowed the throughput to be increased to thousands or even millions of DNA molecules. However, in a typical DNA amplification there are over several billion DNA molecules. This means that even very expensive studies are surveying only a tiny fraction of the total sequence that is present. Conversely, microarray hybridization uses the entire amplicon pool to survey everything that is in the sample. The capability of microarray analysis is especially important when a sample is dominated by several organisms which masks a large number of low abundance organisms including those that may have escaped detection by next-generation sequencing but still play an important role in microbial processes, e.g. pathogens. Indeed, lack on controls and standards and increasing number of ways to take samples, do extractions and use of platforms is increasing the bias in our omics pipelines (Hazen *et al*., 2012).

There are two major thrusts to this grand challenge that will allow us to take advantage of the latest biotechnological breakthroughs in real time. The first is a bioengineering thrust that will take advantage of the large multidisciplinary laboratories in developing key technologies. An example of this is the sample processing experience at US Department of Energy National Labs to develop microfluidics for robust and miniaturized sample processing and microarray hybridization that can be done outside of the laboratory (Liu *et al*., 2011). Miniaturization will reduce reagent costs and increase sensitivity and reaction kinetics for rapid turnaround time. The physical dimensions of a microarray surface will either stay the same or become reduced in size. Microarrays have followed Moore's Law the same as computer speed for exponential increases in probe capacity so that as advances are made in DNA sequencing, the SuperChip would be able to integrate the information for higher resolution analysis. New and evolving technologies will allow us to port the designs for state-of-the-art microarrays today to completely new nanotechnology inspired platforms as they mature.

The second thrust is in bioinformatics to use our existing expertise to take advantage of the rapidly evolving landscape of bioinformatics data. Next-generation DNA sequencing and GC-MS-MS proteomic analysis has resulted in an exponential rise in the amount of publically available DNA and protein sequence data. Through the NIH Human Microbiome Project alone, a $125 million dollar sequencing effort will result in up to one billion new 16S rRNA gene sequences over the next 5 years, the amount of future sequence data will dwarf all current knowledge (http://greengenes.lbl.gov/cgi-bin/nph-index.cgi, http://kbase.science.energy.gov and http://www.microbesonline.org). This increasing capacity of the data set will allow us to resolve microbial species to greatly improved levels and identify functional genes beyond the hypothetical protein level.

Photolithography technology has been used to produce a prototype for this challenge. This Berkeley PhyloChip has been successfully used for the identification and quantification of microbial taxa in environmental and clinical samples to give us an unbiased view of bacterial and archaeal members of complex communities. In less than a day, a sample can be taken and changes identified in microbial community structure over a time-course with replication (Hazen *et al*., 2010). When the high-density Berkeley PhyloChip microarray, with all known DNA sequences encoding 16S rRNA gene sequences, was applied to urban aerosols, the spatiotemporal distributions of known bacterial groups, including specific pathogens, were found to be related to meteorologically driven transport processes as well as sources (Brodie *et al*., 2007). The rapidity, repeatability, comprehensiveness and sensitivity of the PhyloChip for surveying entire bacterial communities in environmental samples suggest that the approach could significantly advance microbial detection and environmental monitoring. A recently updated version [Generation 3 (G3) PhyloChip] is able to categorize prokaryotes (known bacteria and archaeal OTUs) into over 50 000 taxa using 1 100 000 25-mer probes that target variations in the 16S rRNA gene (Hazen *et al*., 2010).

Key features that set the PhyloChip apart from other similar technologies are the use of multiple oligonucleotide probes for every known category of prokaryotic organisms for high confidence detection and the pairing of a mismatch probe for every perfectly matched probe to minimize the effect of non-specific hybridization. A strong linear correlation has been confirmed between microarray probe set intensity and concentration of OUT-specific 16S rRNA gene copy number, allowing quantification in a wide dynamic range. Validation experiments have demonstrated that intensity-based quantification of environmental samples is highly reproducible with variation between replicate chips being less far less than 10% (Hazen *et al*., 2010).

The microarray has been extensively used on complex environmental samples from air, soil and water and the resulting findings have been confirmed by additional methods, including qPCR and 16S rRNA gene clone libraries. LBNL studies using split-samples have confirmed that > 90% of all 16S rRNA sequence types identified by the more expensive clone library method are also identified by the PhyloChip (DeAngelis *et al*., 2011). In addition, the PhyloChip has demonstrated several-fold increases in detected microbial diversity over the clone library method. One of the reasons for this is the high sensitivity of the PhyloChip, with the ability to detect organisms present at a fraction below 10^−4^ abundance of the total sample. Numerous validation experiments using sequence-specific PCR have confirmed that taxa identified by the microarray but not clone libraries were indeed present in the original environmental samples (DeAngelis *et al*., 2011). Although each sample analysis by the PhyloChip provides detailed information on microbial composition, the highly parallel and reproducible nature of this array allows tracking community dynamics over time and treatment. With no prior knowledge, specific microbial interactions may be identified that are key to particular changing environments. The success of this Berkeley PhyloChip in a relatively short time with limited resources demonstrates our capability to address this grand challenge. After demonstrating the utility of this prototype of the eventual ‘SuperChip’ the research community immediately seized on the value of this approach.

Parallel advancements to the PhyloChip have led to breakthroughs in comprehensive fungal species characterization and functional gene analysis of microbes for an even greater understanding of microbial community dynamics. The Berkeley MycoChip uses information from the small subunit rRNA gene sequence, plus the intergenic transcribed spacer (ITS) region and the D1/D2 region of the large subunit rRNA gene as biomarkers to characterize fungal community composition and structure. Due to the paucity of fungal biomarker sequences in public databases it was necessary to create a new fungal sequence database at LBNL to mirror our Greengenes database for bacteria and archaea. Although this microarray is not as far along as the PhyloChip, the prototype arrays have successfully characterized fungal community members in the environment. Work on functional gene microarrays by Dr Zhou's group at the University of Oklahoma has led to the fourth-generation GeoChip to dynamically identify functional activities of multiple members within a community. The most recent version is able to simultaneously distinguish more than 50 000 functional gene variants of more than 400 functional gene categories (Hazen *et al*., 2010; Lu *et al*., 2012).

What is currently lacking is a complete microbial characterization of any sample with no preconceived bias as to what microorganisms are present (Hazen *et al*., 2012). It has already been established that with existing technology we can identify certain classes of microbes to a fine level of resolution. In this grand challenge we propose that the experience and available emerging technologies to create a ‘SuperChip’ in an advanced microarray/lab-on-a-chip type format for the comprehensive identification of all members of a microbial community plus their functional capability, antibiotic resistance and other relevant factors is possible. This will revolutionize the way we study the ‘unseen’ portion of our environment and ourselves with many spinoff benefits, including a better understanding of how microbes influence biogeochemical processes and affect human health. Using recent breakthroughs in microfluidics as a starting point, the goal will be to miniaturize the total assay within the next 5 years to a device that can be taken to the field or placed in a doctor's office. A cheap and portable assay would impact countless areas, including clean water technologies, emerging diseases, bioenergy, infectious disease diagnosis, climate change, food safety, environmental clean-up and bioterrorism. In my opinion it is possible but it will require a very large group of multidiscplenary scientists from multiple institutions crossing many international boundaries and funding over a 5-year period of more than $100 million. Given the impact that this SuperChip could have it is well worth the price.
